# A Case of Nodular Fasciitis Protruding From the Anterior Nostril

**DOI:** 10.7759/cureus.75753

**Published:** 2024-12-15

**Authors:** Shohei Mimura, Terushige Mori, Ryou Ishikawa, Mitsuki Toda, Hiroshi Hoshikawa

**Affiliations:** 1 Otolaryngology, Shodoshima Central Hospital, Takamatsu, JPN; 2 Otolaryngology-Head and Neck Surgery, Kagawa University, Takamatsu, JPN; 3 Diagnostic Pathology, Kagawa University, Takamatsu, JPN

**Keywords:** anterior nostril, myh9-usp6 fusion, myofibroblast sarcoma, nasal cavity, nodular fasciitis

## Abstract

Primary nodular fasciitis of the nasal cavity is quite rare, and only a few cases have been reported. The patient was a 40-year-old man whose chief complaint was a nasal tumor. We suspected fibrosarcoma and operated. The final diagnosis was nodular fasciitis. There was no recurrence at six months postoperatively. In this report, we describe a case of nodular fasciitis protruding from the anterior nostril and reviewed the literature.

## Introduction

Nodular fasciitis is a benign soft tissue tumor of myofibroblast origin that is often misdiagnosed as sarcoma because of its course characterized by rapid growth [1-2]. Recently, detection of the* MYH9-USP6* fusion gene has been found to be diagnostic [3]. Nodular fasciitis was first reported by Konwaler et al. in 1955 as pseudo-sarcomatoid fibromatosis [4]. In 1961, Price et al. proposed the name nodular fasciitis [5]. Tumors are usually less than 20-30 mm in size, and approximately 20% of them occur in the head and neck region, though they are rare in the nasal cavity. In this report, we describe a case of nodular fasciitis that protruded from the right nasal cavity, highlight diagnostic difficulties, and discuss the importance of recognizing rare presentations in otolaryngology.

## Case presentation

A 40-year-old man visited a nearby clinic with a chief complaint of a right nasal tumor that had grown rapidly over the course of a week and begun to protrude through the anterior nostril. In view of the particularly rapid growth, he was referred to a general hospital on the same day. Based on imaging and biopsy findings, fibrosarcoma was suspected, and the patient was referred to our hospital for treatment within one month of his initial visit. The findings at the first visit to the clinic revealed a lobulated neoplastic lesion protruding from the right anterior nostril. The tumor was soft and hemorrhagic. Part of the tumor was already self-destroyed. Blood tests showed no elevation in various tumor markers, such as squamous cell carcinoma antigen (0.9 ng/ml). No other abnormalities were noted.

**Figure 1:**
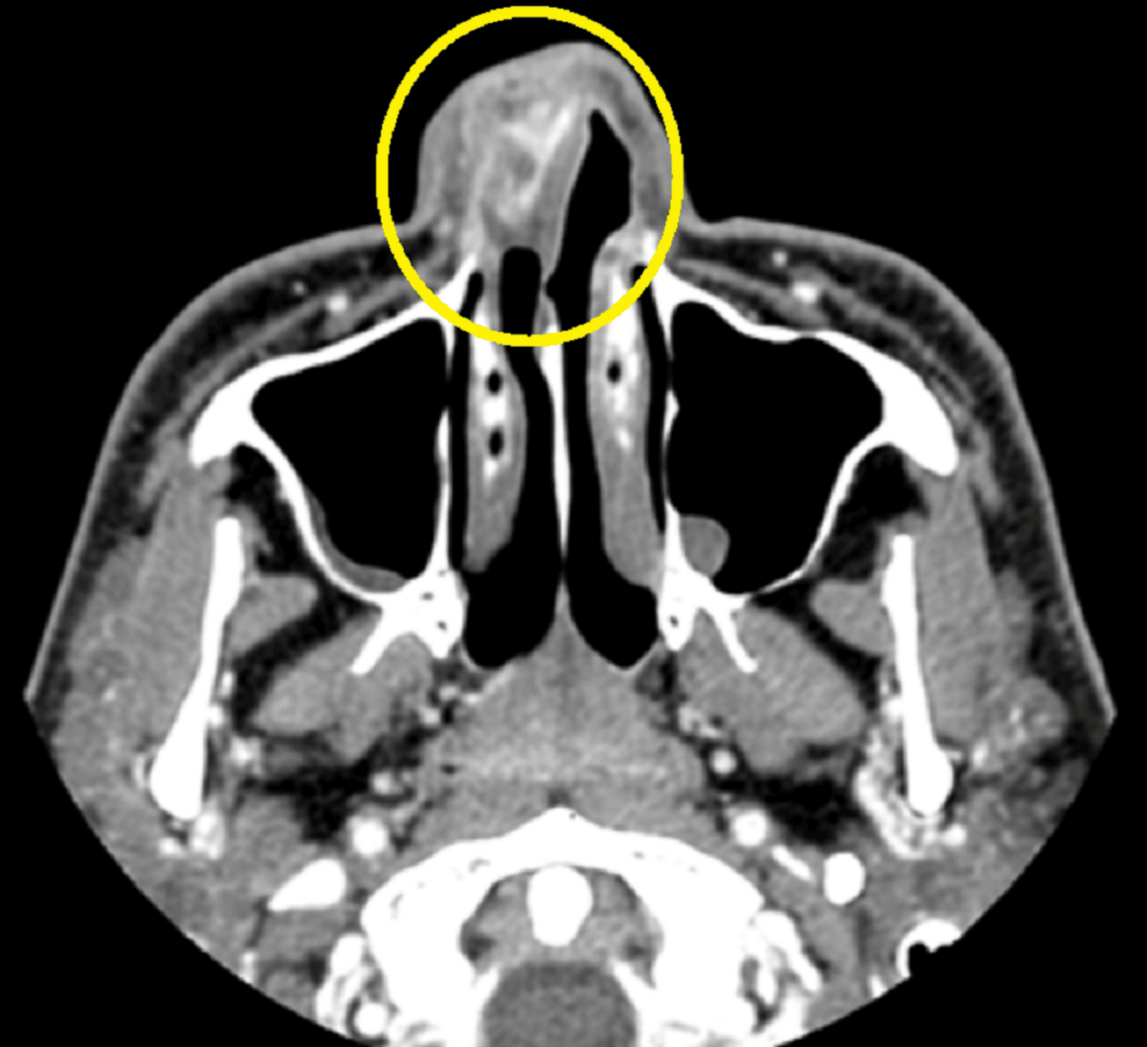
computed tomography. The area enclosed by the circle is the tumor.

Cervical computed tomography (Figure 1) showed a lesion occupying the right nasal vestibule. The inferior nasal dorsum was in contact with the tumor; however, no infiltration was observed. The space-occupying lesion appeared to have a stalk. The borders were relatively well-demarcated, and the interior of the tumor was heterogeneously contrasted.

**Figure 2:**
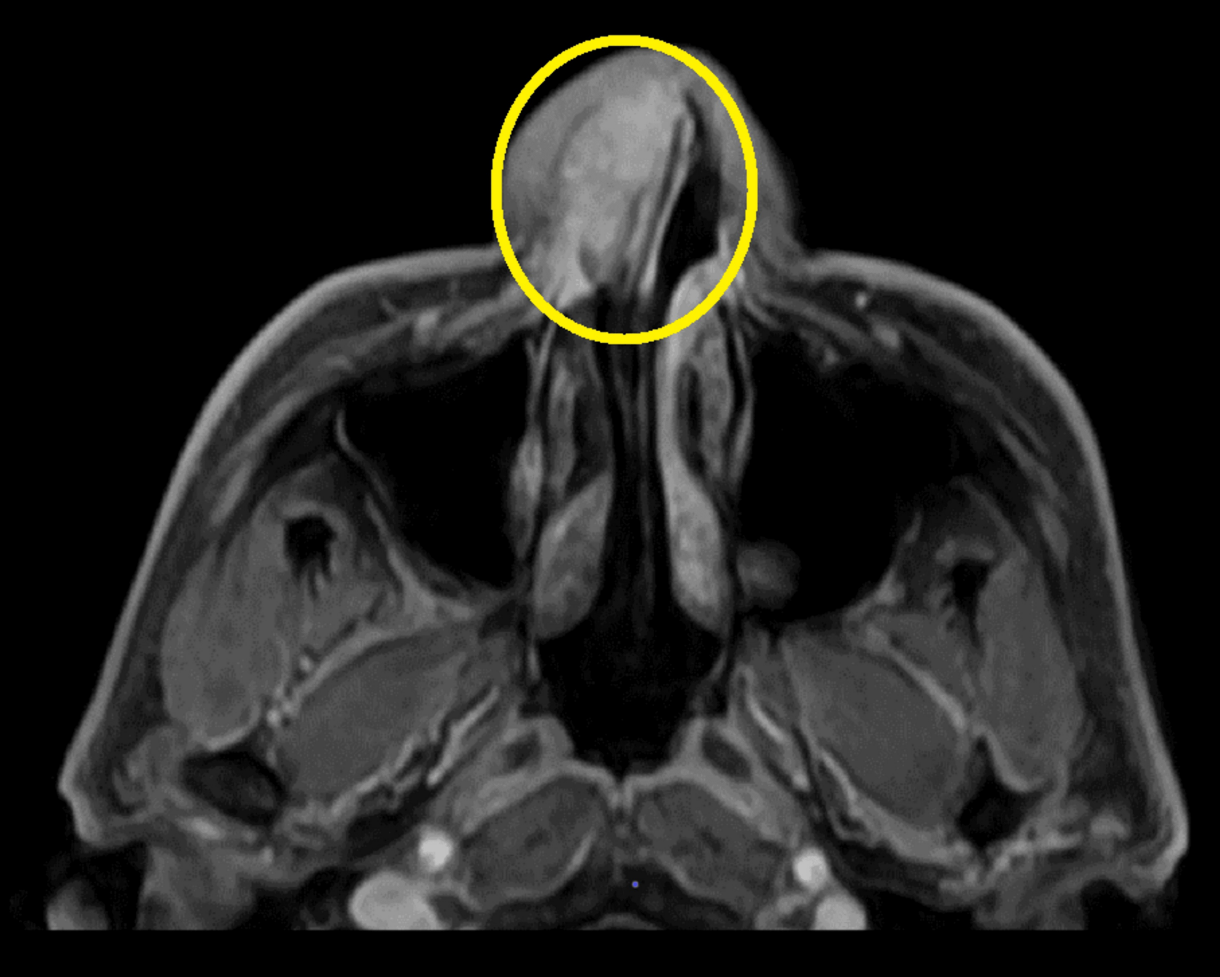
magnetic resonance imaging. The area enclosed by the circle is the tumor.

Cervical magnetic resonance imaging (Figure 2) also revealed a lesion occupying the right nasal vestibule. The space-occupying lesion had a base, and a peripheral branch of the facial artery appeared to emerge and pass through the base. The border was relatively well-demarcated, with a low signal on T1-weighted imaging and a low to slightly high signal on T2-weighted imaging. The tumor showed clear early gadolinium enhancement and prolonged heterogeneous enhancement.

Preoperative pathological examination showed proliferation of tumor cells with spindle-shaped nuclei in a myxoid matrix. The nuclei were irregular, with a fission pattern of approximately eight cells/HPF. Immunohistochemistry revealed that the tumor cells were positive for alpha-smooth muscle actin (αSMA) and negative for S100, CD34, desmin, anaplastic lymphoma kinase (ALK), anti-pan cytokeratin antibody (AE1/AE3), and EMA, and the Ki-67 labeling index was approximately 50%. Based on these findings, a myofibroblastic cell-derived tumor was suspected, and a diagnosis of myxofibrosarcoma or fibromyxoid sarcoma was made.

Based on the above results, the patient was scheduled for removal of a rapidly growing right nasal sarcoma through a lateral nasal incision. Based on the imaging findings, the base of the tumor was thought to be located in the nasal vestibule. Depending on the results of the postoperative histological diagnosis, consideration of additional treatment was planned, such as secondary resection or chemotherapy. Since the tumor was rapidly growing in a young patient and a malignant tumor such as sarcoma was suspected based on the preoperative pathological diagnosis, surgery was performed as soon as possible instead of waiting for the patient to undergo surgery.

**Figure 3:**
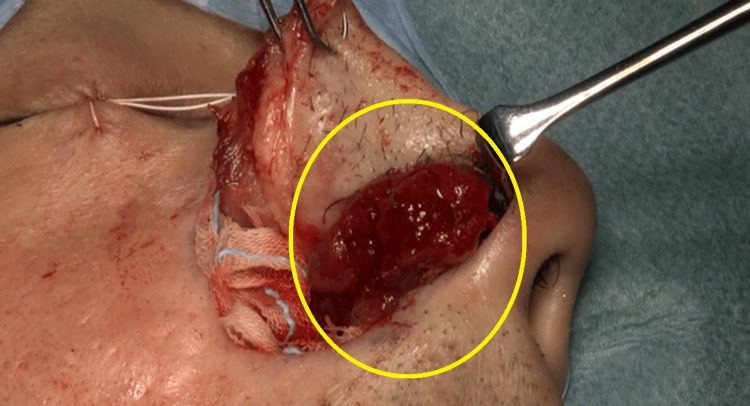
intraoperative findings. Reddish tumor in the center, the base of the lesion was located on the nasal septum at the cutaneous-mucosal transition of the right nasal vestibule.

The patient underwent nasal tumor resection through a lateral nasal incision under general anesthesia. The airway was secured via endotracheal intubation. The surgical field was secured through a lateral nasal incision, and the tumor was clearly visible (Figure 3). The perimeter of the tumor was identified, and as expected before surgery, the base of the lesion was located on the nasal septum at the cutaneous-mucosal transition of the right nasal vestibule. After local injection into the base of the lesion, no deep invasion was observed, and the tumor was resected with the nasal septal mucosa and nasal septal cartilage membrane partially attached. The wound surface was dressed with artificial skin made of collagen, and the lateral nasal incision was sutured. Gauze packings were placed inside the nose to complete the surgery. Since it was judged to be a curative resection, no rapid pathological examination was performed.

**Figure 4:**
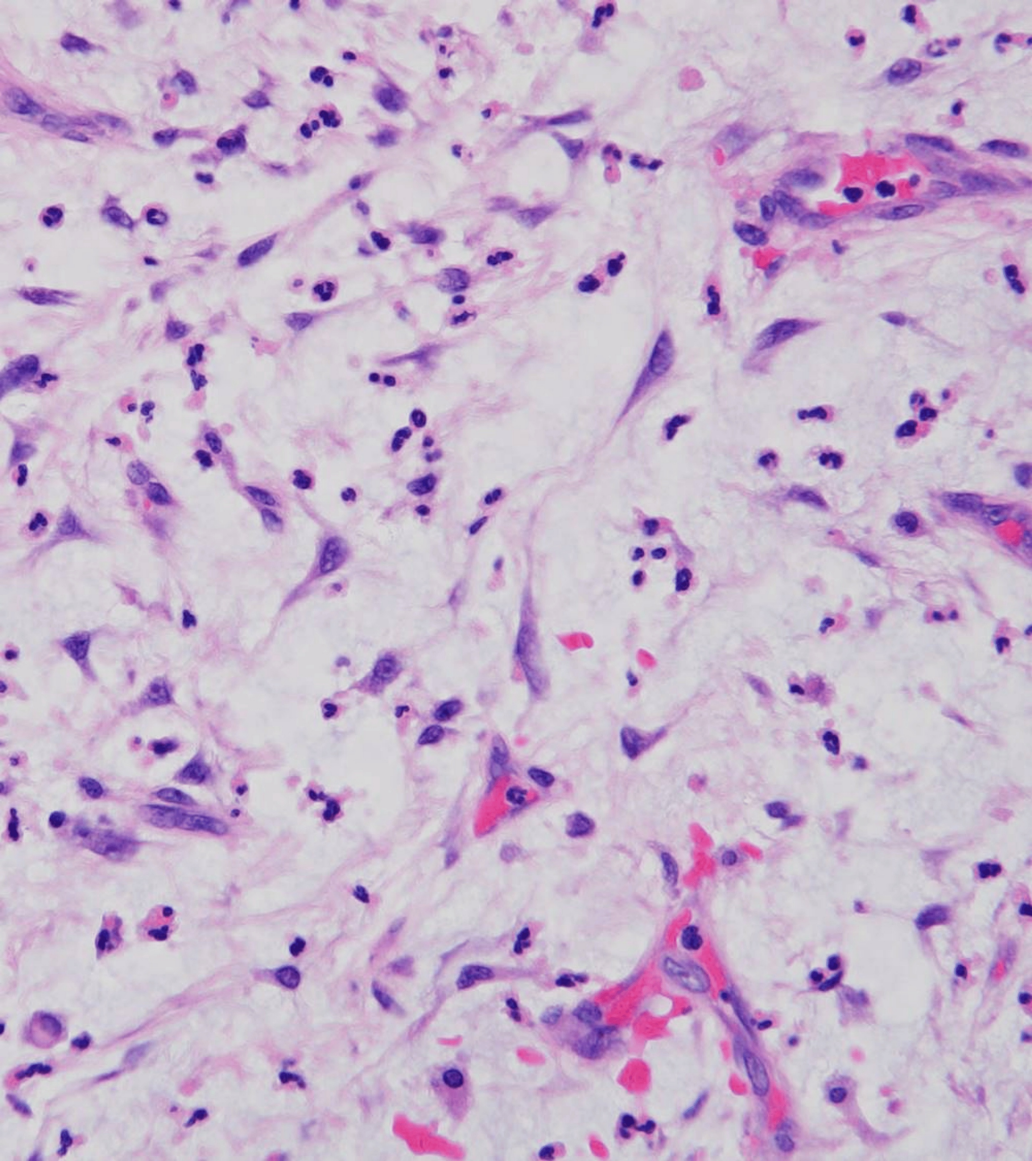
pathological findings. Proliferation of tumor cells in a myxoid matrix, inflammatory cell infiltrates with a mixture of lymphocytes, neutrophils, and histiocytes were observed.

The postoperative pathological examination showed that the excised tumor was a 23×17 mm large lobular mass (Figure 4). There was a proliferation of tumor cells with spindle-shaped to round nuclei in a myxoid matrix. Inflammatory cell infiltrates with a mixture of lymphocytes, neutrophils, and histiocytes were observed. At most, approximately five mitoses/HPF were observed (field of view 22, 40× objective). Immunohistochemical analysis showed the tumor was positive for αSMA and muscle-specific actin (HHF-35) and negative for desmin. The Ki67 labeling index was approximately 30%. The *MYH9-USP6* fusion gene was detected by reverse transcription polymerase chain reaction (RT-PCR)[3]. Based on these findings, nodular fasciitis was diagnosed.

In the postoperative course, wound epithelialization was noted on the postoperative seventh day. The patient was followed up at our outpatient clinic, but there was no recurrence at six months postoperatively.

## Discussion

Nodular fasciitis was first reported by Konwaler et al. in 1955 as “pseudosarcomatoid fibromatosis” and was thought to be a benign reactive fibroblastic proliferation in the subcutaneous soft tissue [4]. In 1961, Price et al. proposed the name “nodular fasciitis” because all cases were located in the fascia or intramuscularly [5]. Previously, the disease was hypothesized to be a response to mechanical stimuli or stress. Recently, however, the involvement of the *MYH9-USP6* fusion gene has been suggested and the tumorigenic aspect of the disease has attracted attention [3]. According to the WHO classification, it is classified as a benign tumor or proliferative lesion belonging to the fibroblast/myofibroblast group [6].

Nodular fasciitis is most prevalent among adults in their twenties to fifties, with no sex differences. The tumor can rapidly increase in size within a few months and sometimes regresses spontaneously. Due to its course characterized by rapid infiltrative growth, it can easily be misdiagnosed as a malignancy, particularly sarcoma [1]. Diagnosis based on imaging findings alone is difficult because the disease lacks characteristic imagining findings [7].

Histologically, careful differentiation is required because of its resemblance to malignant tumors such as lobular tumors and spindle cell carcinoma. The present case showed multidirectional, irregular proliferation of spindle-shaped fibroblasts in a myxomatous fashion. Infiltrates of inflammatory cells, such as neutrophils and lymphocytes, were also observed. Immunohistochemically, αSMA and HHF-35 tended to be positive, whereas CD34, S100, and desmin were negative [8]. Recently, RT-PCR and other methods have shown that *USP6* gene rearrangement is detected in up to 90% of cases, and *MYH9* is identified in approximately 70% of cases, making it useful for the diagnosis of nodular fasciitis [3].

Regarding treatment, local recurrence is not expected to occur following complete resection of the tumor. Surgical resection is therefore commonly performed in such cases. However, several cases of spontaneous resolution have been reported [9,10]. There was also a report that RT-PCR of cytology specimens could be used to confirm USP6 gene rearrangement, and if nodular fasciitis could be diagnosed, follow-up observation could be an option [11].

In practice, surgical resection is the first step in treating this rapidly growing tumor, which can easily be misdiagnosed as sarcoma; however, careful consideration is necessary to avoid overtreatment. If the tumor is even slightly smaller in size, we should suspect nodular fasciitis. As for postoperative follow-up, since there is no possibility of recurrence after curative resection, monthly follow-up for about six months should be sufficient.

## Conclusions

We encountered a case of nodular fasciitis that protruded through the anterior nostril. Nodular fasciitis arising from the nasal cavity is rare. It should be kept in mind that nodular fasciitis can be easily misdiagnosed as a sarcoma. If the tumor shrinks spontaneously while awaiting surgery and a diagnosis of nodular fasciitis is made, surgery can be avoided, but if the tumor is a sarcoma, it can be fatal. Surgical resection is the first step in the treatment of this rapidly growing tumor. Careful consideration is necessary to avoid overtreatment.
